# Patient-derived scaffolds representing breast cancer microenvironments influence chemotherapy responses in adapted cancer cells consistent with clinical features

**DOI:** 10.1186/s12967-023-04806-z

**Published:** 2023-12-20

**Authors:** Maria Carmen Leiva, Anna Gustafsson, Elena Garre, Anders Ståhlberg, Anikó Kovács, Khalil Helou, Göran Landberg

**Affiliations:** 1 Department of Laboratory Medicine, Institute of Biomedicine, Sahlgrenska Academy, Sahlgrenska Center for Cancer Research, University of Gothenburg, 41390 Gothenburg, Sweden; 2 Department of Clinical Pathology, Sahlgrenska University Hospital, 41345 Gothenburg, Sweden; 3 Wallenberg Center for Molecular and Translational Medicine, University of Gothenburg, 41390 Gothenburg, Sweden; 4 Department of Clinical Genetics and Genomics, Sahlgrenska University Hospital, 41345 Gothenburg, Sweden; 5 Department of Oncology, Institute of Clinical Sciences, Sahlgrenska Center for Cancer Research, Sahlgrenska Academy, University of Gothenburg, 41390 Gothenburg, Sweden

**Keywords:** Breast cancer, Patient-derived scaffolds, Cancer microenvironment, Chemotherapy, Doxorubicin, 5-Fluorouracil

## Abstract

**Background:**

The tumor microenvironment clearly influences cancer progressing properties but less is known about how individual cancer microenvironments potentially moderate cancer treatment effects. By cultivating and treating cancer cell lines in patient-derived scaffolds (PDS), the impact of specific characteristics of individual cancer microenvironments can be incorporated in human-like growth modelling and cancer drug treatment testing.

**Methods:**

PDSs from 78 biobanked primary breast cancer samples with known patient outcomes, were prepared and repopulated with donor breast cancer cell lines, followed by treatment with 5-fluorouracil or doxorubicin after cellular adaption to the various microenvironments. Cancer cell responses to the treatments were monitored by RNA-analyses, highlighting changes in gene sets representative for crucial tumor biological processes such as proliferation, cancer stem cell features, differentiation and epithelial-to-mesenchymal transition.

**Results:**

The chemotherapy treatments induced distinct gene expression patterns in adapted cancer cells with clusters of similar treatment responses depending on the patient-derived cancer microenvironment used as growth substrate. The doxorubicin treatment displayed a favorable gene signature among surviving cancer cells with low proliferation (*MKI67*) and pluripotency features (*NANOG*, *POU5F1*), in comparison to 5-fluorouracil showing low proliferation but increased pluripotency. Specific gene changes monitored post-treatment were also significantly correlated with clinical data, including histological grade (*NANOG*), lymph node metastasis (*SLUG*) and disease-free patient survival (*CD44*).

**Conclusions:**

This laboratory-based treatment study using patient-derived scaffolds repopulated with cancer cell lines, clearly illustrates that the human cancer microenvironment influences chemotherapy responses. The differences in treatment responses defined by scaffold-cultures have potential prognostic and treatment predictive values.

**Supplementary Information:**

The online version contains supplementary material available at 10.1186/s12967-023-04806-z.

## Background

Cancer is a heterogeneous group of diseases characterized by an uncontrolled cell growth that can go beyond tissue boundaries, and cancer cells have the potential to spread from the primary site and form metastases [1]. Advances in early detection and cancer treatment have influenced the mortality for some cancer types in parallel with improved quality of life, but there is still substantial risk of therapeutic resistance, disease relapse and metastasis for many cancer patients [2]. Similarly, despite advances in molecular subclassification in breast cancer, clear predictive information of the disease subtype that will respond to specific cancer treatments is still lacking for this most common cancer form in women [3, 4]. Improved treatment predictive systems would therefore be essential in guiding existing and upcoming cancer treatments to increase the precision and selection of patients for each therapy, including conventional chemotherapies. Besides variation in genetic changes in cancer cells, properties of the tumor microenvironments such as oxygenation of cells and acidity have been suggested to affect clinical behaviors as well as responses to cancer treatments [5]. By also providing a physical barrier and altering cell–cell and cell–tumor microenvironment connections, the microenvironment and its extracellular matrix can influence drug responses [6]. Despite the obvious importance of the cancer cell population in cancer progression and clinical aggressiveness, little is known about how the microenvironment-heterogeneity within the cancer niches influences cancer treatment effects. Similar, differences in drug effects on various subtypes of cancer cells including cancer stem cells (CSC) that reside and are linked to different cancer niches, need to be clarified to optimize future treatment strategies and comprehend this complex heterogeneity [7–9].

Modelling the cancer microenvironment in vitro is a major challenge in cancer research and drug discovery. Alternative growth platforms to simplistic 2D-based growth cultures have been developed over the years to recapitulate in vivo-like situations. Cancer organoids and spheroid assays have a more appropriate and 3D-based growth but will not normally include a relevant cancer microenvironment [10]. 3D-printed biomaterials and Matrigel provide important support for cellular 3D growth but are not including a complex and representative cancer microenvironment and lack the patient heterogeneity aspect. In contrast, patient-derived scaffolds (PDSs) created from decellularized primary cancers, maintain the tumor-specific architecture and composition of a human based 3D growth system, and can reveal important properties of patient cancer microenvironments [11, 12]. Breast cancer cell lines adapting to PDSs develop in vivo-like cellular phenotypes, with enrichment in epithelial-to-mesenchymal-transition (EMT) and cancer stem cell features in comparison to monolayer cancer cultures being more proliferative [11, 13]. Clinical features of the original cancer have also been associated with specific gene expression changes in adapting cancer cell lines growing in the different patient-based cancer microenvironments, including differential secretion profiles [14]. Collective data supports that the imprinted information in the cell-free PDSs, represent important information of various cancer progressing properties that can be decoded by the adapting cancer cell lines. The PDS-model can also be used for out of the patient cancer treatment testing, potentially revealing treatment predictive information [11, 13]. Smaller studies of PDSs treated with chemotherapy or endocrine treatments, have shown distinct gene expression changes in adapting cancer cells in relation to the drug treatment, indicating that the PDS-method may be useful tool in determining cancer drug effects in vitro using a human-based model system [15, 16].

Despite the development of new targeted treatment approaches for cancer patients, cytotoxic chemotherapies are highly relevant for several cancer forms and can be administered before and/or after surgery and in combination with other treatments such as immune checkpoint blockade or radiotherapy. Regardless of the combination treatment strategy, there are clear variability in responsiveness and general cytotoxic effects between patients [17, 18]. This study has evaluated the influence of individual cancer microenvironments on drug response, with a focus on standard chemotherapies using PDSs generated from a cohort of breast cancer patients with known clinical characteristics. The results show that the PDS-model can monitor treatment response variations for 5-fluorouracil (5-FU) and doxorubicin (DOX), solely depending on the cancer microenvironment context, further linked to clinical behaviors of the original cancer disease.

## Methods

### Patient material

In this retrospective study, primary invasive tumors were obtained from 78 patients diagnosed between 1980 and 1999 in Western Sweden. The tumor samples were collected from the fresh-frozen tissue tumor Biobank, located at the Sahlgrenska University Hospital Oncology lab (Gothenburg, Sweden). Clinico-pathological characteristics and overall survival data were obtained from the National Quality Registry at the Regional Cancer Center West (Gothenburg, Sweden) and the Cancer Registry at the National Board of Health and Welfare, respectively (Additional file 2: Table S1).

### Tumor decellularization

Primary breast cancer samples were decellularized following previously developed protocols [11, 16]. To obtain the PDS slices used for cell culture, pieces of 6 mm diameter were cut with a biopsy punch needle (Sarstedt), snap frozen in liquid nitrogen and cut to 150 µm slices with a CM3050-S Leica cryotome. Slices were sterilized in peracetic acid 0.1% (Sigma-Aldrich) for 1 h at room temperature, and afterwards washed 24 h in phosphate-buffered saline (PBS; Medicago) supplemented with 1% Antibiotic–Antimycotic (Thermo Fisher Scientific) at 37 °C, 175 rpm. PDSs were stored at 4 °C in a buffer containing PBS, 5 mM EDTA and 0.02% sodium azide until use.

### Cell culture in patient-derived scaffolds

Cell lines were purchased from American Type Culture Collection (ATCC). MCF7 was cultured in Dulbecco’s modified Eagle’s medium (DMEM) supplemented with 10% fetal bovine serum, 1% penicillin/streptomycin, 1% l-glutamine (all from Thermo Fisher Scientific) and 1% MEM Non-Essential Amino Acids (Sigma-Aldrich); whereas MDA-MB-231 was cultured in RPMI-1640 medium supplemented with 10% fetal bovine serum, 1% penicillin/streptomycin, 1% l-glutamine and 1% sodium pyruvate (all from Thermo Fisher Scientific). Culture conditions were 37°C, 5% CO_2_ and humidified atmosphere. Cells were confirmed to be mycoplasma free (Mycoplasma PCR Detection Kit, Applied Biological Materials Inc.) and discarded after 30 passages. For cell culture, PDS slices were washed in PBS for 24 h and then placed in a 48 well plate with 200 µl media. 3 × 10^5^ cells were seeded in 500 µl of cell line specific media supplemented with 1% Antibiotic–Antimycotic (Gibco). After 24 h, PDSs were transferred to a new plate with fresh media, and incubation continued for 21 days. The PDSs were moved to wells with fresh media 1–2 times per week. 64 and 72 PDSs were cultured with MCF7 and MDA-MB-231 cells respectively (58 PDSs overlapped with both cell lines).

### Drug treatments

5-FU (50 mg/ml, Accord) and DOX (2 mg/ml, Actavis) were purchased from Apoteket (Sweden) in a saline solution formulation. Treatment was added after 21 days of cell culture in PDSs, using either 1 mM 5-FU or 15 µM DOX. The effective drug concentrations in PDS cultures were identified in a previous study[15]. After 48 h, treated as well as untreated PDS cultures were harvested in 350 µl RLT buffer (Qiagen) and stored at -80 °C until RNA extraction.

### RNA extraction and cDNA synthesis

The PDS cultures were homogenized with a stainless-steel bead in TissueLyzer II (both Qiagen) for 2 × 5 min at 25 Hrz. The homogenized sample was centrifuged for 3 min at 10,000 rpm using a centrifuge 5417R (Eppendorf) to eliminate cell and PDS debris. RNA extraction was performed using the RNeasy Micro Kit, including a DNase treatment step, in a QIAcube device (all Qiagen) according to the manufacturer’s instructions. The RNA concentration was quantified in a NanoDrop (Thermo Fisher Scientific). Reverse transcription was performed using the GrandScript cDNA synthesis kit (TATAA Biocenter) in the T100 Thermal Cycler (BioRad). The 20 μl reaction volume contained 100–500 ng RNA together with 10,000 RNA Spike II molecules (TATAA Biocenter) for RNA stability control. The temperature profile was 25 °C for 5 min, 42 °C for 30 min, 85 °C for 5 min and cooling at 4 °C. Samples were diluted 1:5 or 1:6 after the synthesis with RNAse free water (Thermo Fisher Scientific).

### Quantitative PCR

Quantitative PCR was performed on CFX384 Touch Real-Time PCR Detection System (Bio-Rad). The 6 μl reaction volume contained 1 × SYBR GrandMaster Mix (TATAA Biocenter), 400 nM of each forward and reverse primer (Additional file 2: Table S2) and 2 μl diluted cDNA. The temperature profile was 95 °C for 2 min, 35–50 cycles of amplification at 95 °C for 5 s, 60 °C for 20 s and 70 °C for 20 s. The melting curve analysis was performed from 65 °C to 95 °C with 0.5 °C/s increments. The evaluated assays were validated with melting curve analyses and agarose gel electrophoresis. Cycle of quantification values were determined by the second derivative maximum method with the CFX Manager Software v.3.1 (Bio-Rad). Quantitative real-time PCR was performed according to the MIQE guidelines[19]. Data was pre-processed with the software GenEx (MultiD) and the target gene expression values were normalized using reference genes identified by the NormFinder algorithm. Gene expressions from PDSs were expressed as relative quantities to the expression of cells grown in 2D conditions, and in base 2 logarithm scale. Drug fingerprints were created for each PDS subtracting untreated log2 values to the treated log2 value for each gene. The drug fingerprints were used for all analyses in this article, unless specified. Data dispersion was calculated using the interquartile range (IQR).

### Statistical analyses

One and two-way ANOVA with Dunnett’s correction for multiple testing was used for comparing treated PDSs to untreated PDSs in GraphPad (Prism). All other statistical analyses were performed in SPSS v.25 (IBM statistics). Pearson correlation analysis was used to analyze correlations between the expressions of different genes. Mann–Whitney U test was performed to assess the association between gene expression and clinico-pathological parameters. Kaplan–Meier method was used to estimate disease-free survival (DFS) curves and log-rank test to compare survival in different strata defined by median, first quartile (Q1, 25%) or third quartile (Q3, 75%). Cox proportional hazards regression analysis was used to evaluate the prognostic impact of gene expression in patients’ survival adjusted for grade, ER (estrogen receptor) status, tumor size, age and lymph node metastasis. Disease-free survival was calculated from the date of diagnosis to the date of recurrence or death caused by breast cancer (defined as endpoint). Univariate analysis was performed using the continuous variable for each one of the genes. The Benjamini–Hochberg method was used for multiple testing correction in the univariate analyses. Multivariable analysis was performed including grade (I and II vs III), ER status, age, tumor size and lymph node metastasis as covariates, and one gene at the time. All the analyses were performed in the entire PDS cohort and *P-*values < 0.05 were considered significant.

## Results

### Patient-derived scaffold cultures with the MCF7 breast cancer cell line show general cytotoxic drug responses

**Fig. 1:**
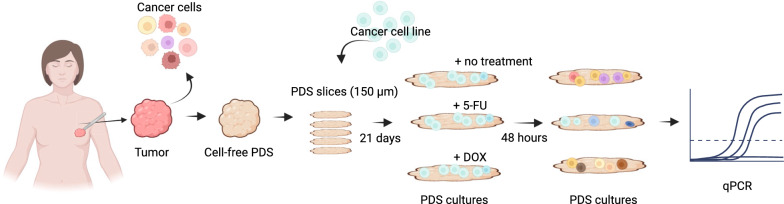
Experimental workflow. Illustration depicting the process from cancer surgery to gene expression analyses of the patient-derived scaffold (PDS) cultures. Primary cancer samples are decellularized to generate PDSs that are cryotome sectioned into 150 µm slices and then used for cancer cell line cultures. After 21 days of adaptation to the patient cancer microenvironment, the PDS cultures are treated with 5-fluorouracil (5-FU) or doxorubicin (DOX) for 48 h followed by gene expression analyses by quantitative PCR. Created with BioRender.com

**Fig. 2:**
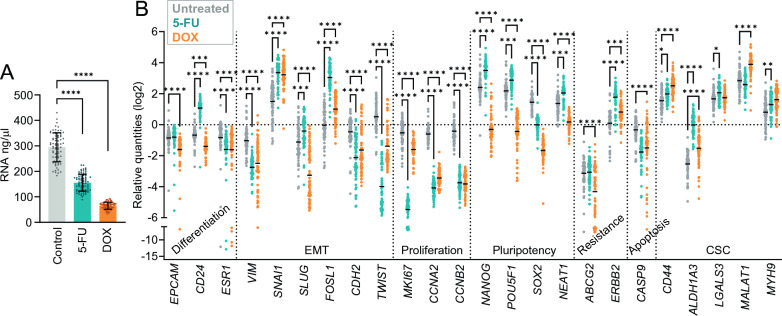
Gene expression changes in MCF7 cancer cells grown in PDSs treated with chemotherapy. **A** RNA yield (ng/µl) of the lysates from patient-derived scaffold (PDS) cultures for controls (untreated), 5-fluorouracil (5-FU) or doxorubicin (DOX) treatment. (****P < 0.0001). **B** Scatter plots showing gene expression levels of untreated and treated PDS cultures, relative to untreated 2D culture gene expression and stablished as 0 value (*P < 0.05, ** P < 0.01. ***P < 0.001. ****P < 0.0001). Gene families for the selected genes are indicated

Cell-free patient-derived scaffolds (PDSs) from 64 patients were seeded with the ER-positive breast cancer cell line MCF7. After 21 days of growth and cellular adaptation, 1 mM 5-FU or 15 µM DOX were added and the PDSs were incubated for an additional 48 h before downstream analysis (Fig. 1; clinico-pathological information in Additional file 2: Table S1) [15]. Untreated PDS-cultures for each patient as well as 2D-cultures were included as controls. The RNA yield from the PDS-cultures, used as an indirect quantification of cell numbers, decreased up to 50% with 5-FU treatment and 75% for DOX treatment compared to untreaded PDS controls (Fig. 2A). The RNA levels in the different PDS-cultures further varied and the total amount of RNA after the two treatments correlated (*r* = 0.6, *P* < 0.0001, Additional file 1: Fig. S1A), whereas RNA yields of untreated PDS and 5-FU or DOX treatment did not correlate (Additional file 1: Fig. S1B, C). These data suggested that the number of cells in the PDSs before treatment did not influence the treatment responses but that individual PDS-cultures with adapted cancer cells responded similarly to both drugs.

Targeted gene expression in PDS-cultures after 5-FU and DOX treatment were assessed by qPCR using 24 selected marker genes involved in different cancer-related processes (Additional file 2: Table S2). Untreated PDS-cultures showed PDS-specific effects on the gene expression but with general decrease of proliferation and differentiation associated genes and increase of pluripotency and CSC genes compared to reference 2D cultures (Fig. 2B). Parallel PDSs treated with 5-FU or DOX, displayed a pronounced downregulation of the three proliferation markers (*MKI67, CCNA2* and *CCNB2*), several EMT markers (*VIM, CDH2* and *TWIST*), the differentiation marker *ESR1*, the pluripotency marker *SOX2* and the apoptosis gene *CASP9* compared to untreated PDSs. Both drug treatments also induced increased expression of several CSC markers (*CD44, ALDH1A3* and *MYH9),* EMT markers *SNAI1* and *FOSL1,* and the resistance gene *ERBB2* (Fig. 2B). The main differences for the two treatments were observed for the pluripotency markers *NANOG*, *POU5F1* and *NEAT1*, which were higher after 5-FU treatment but lower after DOX. 5-FU treatment further caused increased levels of the EMT markers *SLUG* and differentiation marker *CD24,* in contrast to DOX treatment. Altogether, the two drugs as well as the PDS in itself, clearly influenced the gene expression of the remaining cancer cells after treatment, supporting that individual cancer microenvironment, provided by the PDS, affected the treatment response.

### Specific cancer microenvironments influence the expression of genes related to various key tumor biological processes similarly after treatment with 5-fluorouracil or doxorubicin

**Fig. 3:**
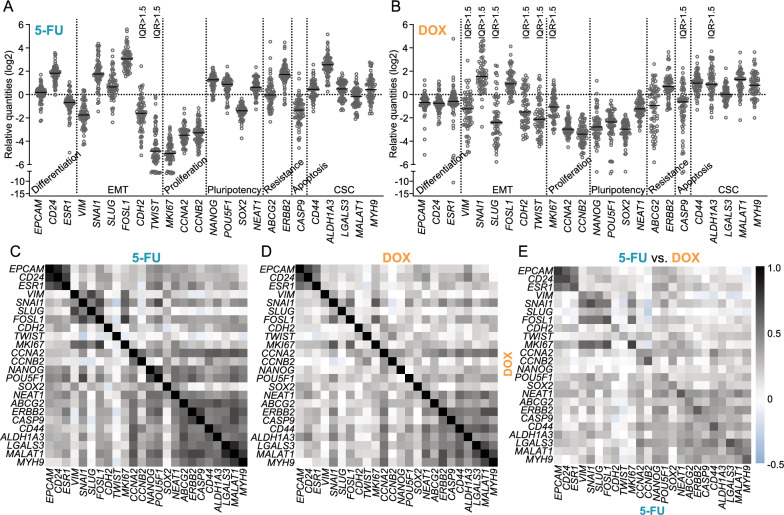
5-fluorouracil and doxorubicin induce similar changes in differentiation and EMT genes in individual MCF7 PDSs. **A**, **B** Drug fingerprints of patient-derived scaffold (PDS) cultures obtained subtracting the untreated gene expression value from the treated value for each gene after treatment with 5-fluorouracil (5-FU) (**A**) or doxorubicin (DOX) (**B**). The largest variation in gene expression changes between individual PDSs is indicated by interquartile range > 1.5 (IQR). **C**, **D**, **E** Pearson correlation analyses between the drug fingerprints for each gene in the individual PDSs treated with 5-FU (**C**), DOX (**D**) or 5-FU versus DOX (**E**). (P-values for each analysis are detailed in Additional file 2: Tables S3–5)

We next outlined the specific drug effect for each PDS separately. The “drug fingerprint” for each PDS was obtained by subtracting the gene expression levels of untreated PDS from the corresponding treated PDS, as presented in Fig. 3A, B. The EMT genes *TWIST* and *CDH2* showed the largest differences in expression levels for 5-FU fingerprint data with interquartile range > 1.5 (IQR log2, Fig. 3A). Similarly, DOX-treatment induced more distinct PDS-specific responses in most EMT genes (*TWIST, VIM, SNA1, CDH2* and *SLUG,*) as well as for *MKI67, ABCG2* and *CASP9* (Fig. 3B).

All 5-FU drug fingerprint data, indicated a strong positive correlation (*r* > 0.6, *P* < 0.05) between differentiation genes (*EPCAM, CD24* and *ESR1)*, as well as several EMT genes (*VIM, SNAI1, SLUG* and *FOSL1*) (Fig. 3C and P-values in Additional file 2: Table S3). Similar associations were observed for proliferation markers (*CCNA2* and *CCNB2)* and pluripotency genes (*NANOG* and *POU5F1*). A large cluster of genes with comparable behaviours included the CSC markers (*CD44*, *ALDH1A3, MYH9*, *MALAT1* and *LGALS3*), the apoptosis marker *CASP9*, and chemotherapy resistance-related markers *ABCG2* and *ERBB2*. Interestingly, DOX fingerprint showed corresponding but less pronounced correlation patterns as for 5-FU (Fig. 3D and P-values in Additional file 2: Table S4). When comparing the 5-FU and DOX treatment drug fingerprints, many individual genes showed striking and similar PDS-dependent drug responses (*r* > 0.6, *P* < 0.05) including *EPCAM*, *CD24, ESR1, SNAI1, FOSL1, MKI67, CCNB2, NEAT1, ERBB2, CD44, LGALS3* and *MYH9* (Fig. 3E and P-values in Additional file 2: Table S5). Common responses for 5-FU and DOX treatments were also observed for genes within the same gene families or with similar functions (*r* > 0.5, *P* < 0.05), such as differentiation (*EPCAM, CD24* and *ESR1*) and EMT (*VIM, SNAI1, SLUG* and *FOSL1*). In contrast, genes involved in CSC, drug resistance and apoptosis showed strong correlation for each treatment separately but were not significantly associated when comparing the two treatments.

### Drug response profiles of patient-derived scaffold cultures correlate with clinical features of the original cancer

**Fig. 4:**
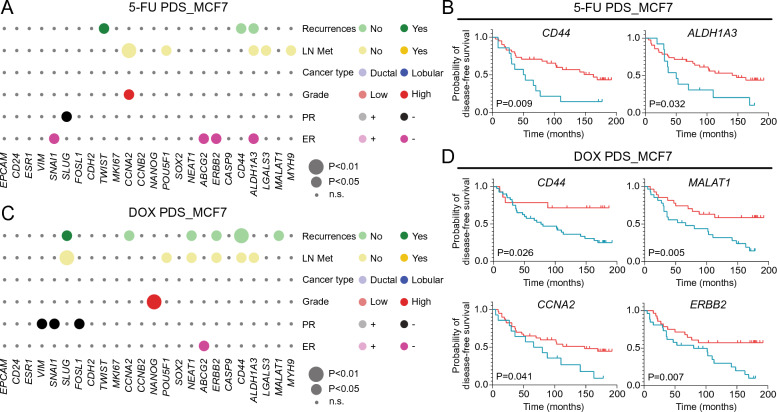
Specific gene expression changes in the MCF7 PDSs drug fingerprint are linked to original cancer characteristics. **A**, **C** Bubble plots representing the association between changes in gene expression in treated patient-derived scaffolds (PDSs) with 5-fluorouracil (5-FU) (**A**) or doxorubicin (DOX) (**C**) and clinico-pathological data of the original cancer (LN Met—lymph node metastases, PR—progesteron receptor status, ER—estrogen receptor status). The circle size represents the level of significance (P-value) and the circle color indicates the clinical variable (y axis) that was associated with higher gene expression levels in PDSs (x axis). For detailed information, see Additional file 2: Table S6 and Additional file 1: Fig. S2 and S3. **B**, **D** Kaplan–Meier plots displaying the relationship between disease-free survival (DFS; blue, low expression; red, high expression) and *CD44* and *ALDH1A3* drug fingerprints after 5-FU treatment (**B**) or *CD44*, *MALAT1*, *CCNA2* and *ERBB2* drug fingerprints after DOX treatment (**D**). P < 0.05, log-rank (detailed information in Additional file 2: Table S8)

Next, we examined if the PDS-drug fingerprint values were associated with clinical patient features of the original cancer disease as summarized in Additional file 2: Table S1. For the 5-FU treatment, several significant associations were observed with notable links between clinico-pathological data and stemness/pluripotency as well as drug resistance related genes (Fig. 4A, Additional file 2: Table S6 and Additional file 1: Fig. S2). Increased expression of the resistance genes *ABCG2* and *ERBB2,* the CSC gene *ALDH1A3,* as well as the EMT gene *SNAI1* after 5-FU treatment in PDSs were linked to ER-negative status of the primary cancer. Higher expression of the EMT gene *SLUG* was also associated with PR (progesterone receptor)-negativity. On the other hand, lower expression of the CSC-associated genes *ALDH1A3*, *LGALS3* and *MYH9* and the pluripotency gene *POU5F1* assessed by fingerprint values, were instead associated with the presence of lymph node metastases (LN Met). Pronounced downregulation of the proliferation gene *CCNA2* in 5-FU treated PDSs, was also associated with lymph node metastases as well as low tumor grade. Interestingly, several drug fingerprint values were associated with disease recurrences, as downregulation of CSC genes *ALDH1A3* and *CD44* or upregulation of the EMT gene *TWIST* after treatment (Fig. 4A). *CD44* and *ALDH1A3* expression changes after 5-FU treatment were also significantly linked to disease-free survival using univariate and multivariable Cox regression and Kaplan–Meier analyses (*P* < 0.05), as illustrated in Fig. 4B (Additional file 2: Table S7 and Additional file 1: Fig. S4).

DOX treatment PDS fingerprint values for resistance, EMT, stemness and pluripotency genes were also associated with several clinical variables (Fig. 4C, Additional file 2: Table S6 and Additional file 1: Fig. S3). As observed for 5-FU treatments, PDSs mediating higher levels of the drug resistance gene *ABCG2* after DOX treatment were linked to ER-negative status. Breast cancer with lymph node metastases were in general associated with lower levels of the CSC genes *ALDH1A3* and *CD44*, pluripotency markers *POU5F1* and *NEAT1*, EMT marker *SLUG*, and drug resistance associated gene *ERBB2,* after DOX treatment. A less marked downregulation of *NANOG* expression after DOX treatment was also significantly linked to high grade cancer. Several EMT markers (*VIM*, *SNAI1* and *FOSL1*) were associated with PR-status, where higher expression levels after treatment were linked to negative PR-status. Interestingly, several DOX-treatment induced gene expression changes in PDSs were also linked to disease-recurrences, as lower levels of CSC genes *CD44* and *MALAT1*, pluripotency marker *NEAT1*, drug resistance associated gene *ERBB2* and proliferation gene *CCNA2,* while the opposite direction was observed for the EMT marker *SLUG* (additional univariate and multivariable Cox regression data in Additional file 2: Table S7). A less pronounced reaction to the DOX-treatment was further significantly linked to disease-free survival for CSC genes *CD44* and *MALAT1* and the chemoresistance gene *ERBB2,* whereas clear downregulation of the proliferation gene *CCNA2* was significantly associated with poor disease-free survival (chi-square, *P* < 0.05, log-rank, *P* < 0.05) (Fig. 4D, Additional file 1: Fig. S4).

Altogether, we observed drug specific changes in the PDS-cultures but importantly also mutual links between 5-FU and DOX treatment responses and associations to clinical features of the original breast cancer disease. The results clearly indicated that cancer microenvironments mediating fewer gene changes, including CSC-markers, after chemotherapy treatment of adapted MCF7 cells, were associated with increased risk of metastatic-disease or breast cancer-related death.

### Treatment responses of ER-negative cell line adapted to patient-derived scaffolds partially overlap with ER-positive cell line data, confirming varying effects of drug responses in different cancer microenvironments

**Fig. 5:**
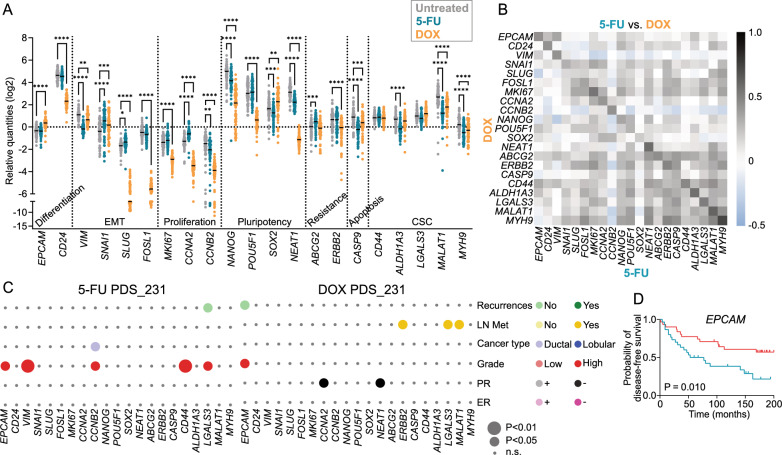
Drug fingerprint of MDA-MB-231 cells cultured in PDSs in relation to clinical characteristics. **A** Scatter plot showing the gene expression for 72 patient-derived scaffolds (PDSs) cultures with MDA-MB-231 cancer cells treated with 5-fluorouracil (5-FU) or doxorubicin (DOX) for 48 h in comparison to untreated PDS cultures. Data is relative to gene expression in untreated 2D culture (*P < 0.05. ** P < 0.01. ***P < 0.001. ****P < 0.0001). **B** Heatmap depicting Pearson correlation coefficients (ρ-values) for the individual gene expression changes in the drug fingerprints for the PDSs treated with 5-FU versus DOX. (P-values for each analysis are detailed in Additional file 2: Tables S8). **C** Bubble plots representing the association between changes in gene expression in treated PDSs with 5-FU or DOX to clinico-pathological data of the original cancer (LN Met—lymph node metastases, PR—progesteron receptor status, ER—estrogen receptor status). The circle size represents the level of significance (P-value) and the circle color indicates the clinical variable (y axis) that was associated with higher gene expression levels in PDSs (x axis). (For detailed information, see Additional file 2: Tables S11 and Additional file 1: Fig S7. **D** Kaplan–Meier plot displaying the relationship between *EPCAM* drug fingerprint after DOX treatment (blue, low expression; red, high expression) and disease-free survival. P < 0.05, log-rank (detailed information in Additional file 2: Table S11)

As previously described, the analysis of different cancer cell lines in PDS cultures have the potential of consolidating associations between PDS-dependent gene expression changes and clinical features of the tumors as well as uncovering new links [13]. The triple-negative breast cancer cell line MDA-MB-231 was therefore cultured in 72 PDSs, resulting in 58 overlapping PDS-cultures with MCF7 cells. As reported previously [13], common changes in untreated MCF7 and MDA-MB-231 cancer cell lines grown in PDS cells were decreased expression of proliferation genes (*MKI67, CCNA2* and *CCNB2*), and a profound increase in pluripotency markers (*NANOG, POU5F1, SOX2* and *NEAT1*) as well as CSC markers (*CD44, LGALS3* and *MALAT1*) compared to 2D cultures (Figs. 5A and  2B).

When adding treatments to the MDA-MB-231 PDS-cultures, RNA levels decreased in the same proportion as for MCF7 PDS-cultures (around 50% and 75% for 5-FU and DOX treatments respectively) although the initial RNA yield of MDA-MB-231 cells seemed to influence the treatment effect (Additional file 1: Fig. S5). Moreover, 5-FU treatment only induced minor changes in most of the analyzed genes compared to treated MCF7 PDS-cultures, and the pluripotency genes *NEAT1* and *NANOG* and the CSC marker *MYH9* were further influenced in opposite direction (Fig. 5A). In contrast to 5-FU, MDA-MB-231 cells growing in PDSs and treated with DOX demonstrated a marked response with noticeable reduction of several markers for differentiation (*EPCAM*), EMT (*SLUG* and *FOSL1*), proliferation (*MKI67*, *CCNA2* and *CCNB2*) and pluripotency (*NANOG*, *POU5F1* and *NEAT1*) (Fig. 5A). When comparing the two treatments and cell lines, DOX treatment substantially decreased both proliferation and pluripotency genes in both cell lines while 5-FU mainly decreased proliferation, supporting a more ideal and broader treatment effect for DOX compared to 5-FU in the PDS-cultures.

5-FU drug fingerprints for MDA-MB-231 PDS-cultures showed in general low inter-scaffold specific variability (IQR > 1.5) for all the examined genes (Additional file 1: Fig. S6A). DOX treatment nevertheless induced larger and more divergent responses for the EMT markers *SNAI1* and *SLUG,* the pluripotency markers *NANOG* and *SOX2* as well as the CSC marker *MALAT1* (Additional file 1: Fig. S6B). When comparing the drug fingerprints for 5-FU and DOX, several significant associations between individual genes were observed (*r* = 0.4–0.6, *P* < 0.05), suggesting some similarities of the effects of the two treatments in MDA-MB-231 PDS-cultures, but the results were less distinct compared to MCF7 cells (Fig. 5B, P-values in Additional file 2: Table S9). Correlations were observed for genes involved in differentiation (*EPCAM* and *CD24*), the EMT marker *VIM*, proliferation markers (*MKI67, CCNA2* and *CCNB2*), the pluripotency gene *NEAT1*, resistance associated genes (*ABCG2* and *ERBB2*) as well as CSC markers (*CD44*, *ALDH1A3* and *MYH9, r* > 0.7). Genes involved in similar processes often showed comparable drug fingerprint changes after both treatments, including a large subgroup of positively correlated genes containing CSC-related and resistance markers. However, when comparing the treatment responses for the two cancer cell lines, there were not strong associations between the drug fingerprints suggesting that the two cancer cell lines reacted rather diverse to the treatments in the PDS despite showing some similarities for individual PDS responses (Additional file 1: Fig. S6C, Additional file 2: Table S10).

In general, MDA-MB-231 treated PDS-cultures showed fewer associations between individual gene changes and clinical characteristics compared to treated MCF7 PDS-cultures. Among the clinical variables, tumor grade was nevertheless more often associated with gene changes after 5-FU treatment in MDA-MB-231 cells, whereas DOX treatment showed additional associations mainly with lymph node metastasis (LN Met) (Fig. 5C, Additional file 2: Table S11, Additional file 1: Fig. S7). Regarding potential prognostic impact of the MDA-MB-231 PDS-drug fingerprints, low *EPCAM* expression after treatment was associated with poor disease-free survival in both multivariable Cox regression analyses (*P* < 0.05) and Kaplan–Meier analyses (*P* < 0.05) (Fig. 5D, Additional file 2: Table S12 and 13). Overall, MDA-MB-231 PDS-cultures showed less varying influence from the cancer microenvironment in relation to 5-FU treatment, but indeed a pronounced and variable effect of DOX treatment. Triple-negative breast cancer MDA-MB-231 cells growing in PDSs also showed less distinct associations to clinical properties of the original cancer in comparison to the hormone positive and more differentiated MCF7 cells.

## Discussion

In this study, we utilized a human-based cell-free scaffold growth model to study the drug response of two breast cancer cell lines, MCF7 and MDA-MB-231, adapted to more than 60 different cancer microenvironments using two frontline chemotherapeutic agents. The results indicated that the cancer cells showed both similar response patterns after treatment as well as clear variations depending on the cancer microenvironment and tumor biological processes analyzed. Interestingly, many of the gene expression changes observed in the remaining cancer cells after treatment were linked to clinical characteristics of the original cancer disease, clearly validating the importance of the cancer microenvironment in influencing treatment responses and clinical behaviors.

In line with previous studies, the growth of the cancer cells in various PDSs displayed differential cellular adaptations as a response to the individual cancer microenvironments, showed by the large inter-scaffold variation in gene expression for both MCF7 and MDA-MB-231 cancer cells [11, 13]. Despite this underlying variation between the PDS-cultures, there were additional changes in gene expression after treatment with chemotherapies 5-FU and DOX. The most pronounced variation in gene expression between PDSs after treatments were observed for genes related to EMT. The acquisition of mesenchymal features by epithelial cells is important for biological processes as embryogenesis and wound healing but is also critical in tumorigenesis and especially, in mediating infiltrative and metastatic behaviors [20–23]. Intercalating agents, such as DOX can affect invasion and proliferation of cancer cells, as well as degradation of extracellular matrix by MMPs (matrix metalloproteinases) through the upregulation of the EMT transcription factor *SNAI1* [24]. The data presented in this study using PDS-cultures also indicated an upregulation of *SNAI1* after DOX treatment for both cell lines as well as large variation in the important EMT regulators *TWIST* and *SLUG* and mesenchymal markers *CDH2* and *VIM*.

Interestingly, PDS-cultures showed many similarities in the responses to 5-FU and DOX treatments. When detailing the RNA levels of cancer cells grown in PDS as a surrogate marker for number of cells, there was a significant association between the 5-FU and DOX treated PDSs, indicating that the individual cancer microenvironment influenced the cytotoxic and antiproliferative effects of the two chemotherapies similarly. Besides, many gene fingerprint values involving important tumor biological processes correlated for the two treatments, supporting that the properties of individual PDSs led to similar selective killing of subsets of cancer cells for both chemotherapies. These results indicate that individual properties of the cancer microenvironments provided by the PDSs differently protect the cancer cells from the drugs, alternatively influencing drug permeability or accessibility to the fraction of more treatment resistant cell subpopulations such as cancer stem cells. Alterations in cancer cell phenotypes as well as increased stemness have been linked to chemoresistance, as well as characteristics of the cancer niches such as stiffness and biomechanical properties of the extracellular matrix influencing cancer cell differentiation [25, 26]. Patient-derived scaffolds have different mechanistic properties and protein compositions, which may influence drug transfusion along with specific killing [11]. Chemotherapies such as 5-FU and DOX can also influence cell-ECM connections through focal adhesions and integrin signaling in cells as well as anti-apoptotic signaling, potentially contributing to the varying gene expression monitored in the PDS models [27, 28]. In this study, the effect on CSC-features defined by both CSC and pluripotency genes showed profound differences for cancer cells surviving the treatments for the two breast cancer cell lines. Both treatments increased the levels of CSC associated genes, but DOX-treatment led to marked decreased gene expression of all pluripotency genes, whereas 5-FU increased three out of four genes. This suggests that DOX also target pluripotent cells and not only the proliferative subpopulation in this human-based growth model system, whereas 5-FU was less efficient in limiting CSC defined by the presence of pluripotency genes. Despite a general marked decrease of pluripotency by DOX, there was still a large variation in the response between the patient scaffolds, supporting the importance for the cancer microenvironment in influencing chemotherapy effects of key cancer properties as cancer stemness and pluripotency.

Published studies clearly support that the cancer stroma contains clinically relevant information about disease behaviors, as well as potential drivers of malignant behaviors and predictive information for treatment responses [7, 29–31]. This critical imprinted information can be decoded by direct measurements of the microenvironment alternatively via functional testing as presented here using the PDS-model [32–34]. We have earlier shown that the proteomic composition of cell-free PDS can be linked to clinico-pathological data as histological grade and proliferation status of the original cancer [11]. In addition, gene expression changes in cancer cells adapted to the PDSs were linked to clinical features, such as ER, PR, grade, and aggressiveness of the cancer [12, 13]. Here, we also observed links between clinical cancer properties and induced gene expression in cancer cells growing in PDSs after chemotherapy treatments. Lymph node metastasis and recurrences were the clinical parameters most often associated with the drug fingerprints in responses to chemotherapy. Interestingly, similar changes involving the same genes or genes within a family, and predominantly CSC and pluripotency features, were associated with clinical parameters. Lymph node metastasis is an important clinical parameter, not fully comparable with distant metastases but still linked to aggressive disease as cancer cells can establish cancer growth in nearby lymph nodes [35, 36]. For example, the breast CSC marker *ALDH1A3* was associated with lymph node status in both 5-FU and DOX treated MCF7-PDSs. Similarly, aldehyde dehydrogenase 1 (ALDH1) expression in cancer cells has been link with lymph node metastasis in many cancer types [37]. In line with these observations, changes in expression of several CSC markers after either 5-FU or DOX treatment were associated with disease-free survival. Surprisingly, an increased expression of *CD44*, *ALDH1A3* and *MALAT1* after treatments in MCF7 PDS-cultures were associated with better outcome. The activity of ALDH1 is mainly due to the isoform *ALDH1A3* and has been associated with metastatic disease in cancer as well acquired chemoresistance in colon cancer [38, 39]. In contrast, in vitro studies using *ALDH1A3*-silenced breast cancer cell lines showed increased migratory capacity along with decreased colony/metastasis formation capacity supporting the data presented in this study [40]. Besides, high expression of *CD44* in breast cancer has been associated with improved survival after neoadjuvant chemotherapy treatment [41]. However, the exact importance and relevance for the various marker and regulators associated with CSC as well as pluripotency, might vary between different cancer cell lines, potentially influencing the presented results. The microenvironment from highly malignant and progressing cancer cases might also mediate a limited capacity to respond to the treatments, mirrored by the direction of the responses of the pluripotency markers as well as several other tumor properties. As detailed previously using different cell lines, the tumor microenvironment provided by the PDS induces similar but also cell line dependent adaptations, uncovering associations between growth influencing properties in PDSs and aggressive features of the original tumor [13]. In line with these findings, expression changes in the CSC markers in MDA-MB-231 as well as MCF7 cells growing in PDSs and treated with DOX, were associated with lymph node metastasis. The addition of MDA-MB-231 cancer cells in PDS cultures further uncovered additional information related to the aggressiveness of the disease such as associations between specific gene changes and high-grade cancer. Altogether, our results indicate that the PDS model can help to identify those patient cancer microenvironments that might support resistant cancer cell subpopulations that will not be targeted by the conventional treatments, implying a potential risk of recurrences and metastasis. This might be a useful information when deciding a treatment combination including for example cancer stem cell target drug or when testing drugs in development phases.

## Conclusions

This comprehensive study of a large cohort of breast cancer PDS repopulated with donor cancer cell lines treated with chemotherapies, demonstrate that clinical treatment studies can be performed in the laboratory using the patient scaffold, and still monitor patient-specific differences in responses to the treatment solely based on the varying microenvironment. The PDS-model further produced clinically relevant data, and several gene expression changes after treatment were linked to patient survival, suggesting that the functional test of the cancer microenvironment mirrored both prognostics, as well as potential treatment predictive information. The possibility to decode key tumor progressing properties using PDS, including cancer treatment responses, will motivate future and larger drug discovery and precision medicine focused studies using human-derived cancer scaffolds.

### Supplementary Information

**Additional file 1: Fig. S1.** RNA yield in MCF7 patient-derived scaffold lysates. **Fig. S2**. Significant associations between 5-flourouracil drug fingerprints and clinical characteristics of the original tumors. **Fig. S3.** Significant associations between doxorubicin drug fingerprints and clinical characteristics of the original tumors. **Fig. S4.** Kaplan–Meier plots displaying the relationship between disease-free survival and drug fingerprints in patient-derived scaffolds. **Fig. S5.** RNA yield in MDA-MB-231 patient-derived scaffold lysates. **Fig. S6.** Drug fingerprints of patient-derived scaffold cultures with MDA-MB-231. **Fig. S7.** Scatter plots for clinical characteristics versus MDA-MB-231 drug fingerprints.

**Additional file 2: Table S1.** Patient and clinico-pathological characteristics of the cohort used for creating patient-derived scaffold cultures with MCF7 or MDA-MB-231. **Table S2.** Primer list. **Table S3.** P-values of the Pearson correlation analyses between the drug fingerprints for each gene in the individual MCF7 PDSs treated with 5-FU and plotted Fig. 3C. **Table S4.** P-values of the Pearson correlation analyses between the drug fingerprints for each gene in the individual MCF7 PDSs treated with DOX and plotted in Fig. 3D. **Table S5.** P-values of the Pearson correlation analyses between the drug fingerprints for each gene in the individual MCF7 PDSs 5-FU versus DOX and plotted in Fig. 3E. **Table S6**. Median values and P-values of Mann–Whitney U comparison between gene expression in MCF7 patient-derived scaffolds and clinical parameters. **Table S7.** Univariate and multivariable cox-regression analyses of gene expression data from MCF7 cells grown in the patient-derived scaffolds following treatment with 5-fluorouracil or doxorubicin. **Table S8.** Log-rank tests of the Kaplan–Meier plots illustrating disease free survival in patient-derived scaffolds cultured with MCF7 and treated with 5-fluorouracil and doxorubicin. **Table S9**. P-values the Pearson correlation analyses between the drug fingerprints for each gene in the individual MDA-MB-231 PDSs 5-FU versus DOX and plotted in Fig. 5B. **Table S10.** P-values of the Pearson correlation analyses between the MCF7 and MDA-MB-231 drug fingerprints for each gene in the individual PDSs treated with 5-FU (top) or DOX (bottom) and plotted in Fig. S6C. **Table S11.** Median values and P-values of Mann–Whitney U comparison between gene expression in MDA-MB-231 patient-derived scaffolds and clinical parameters. **Table S12.** Univariate and multivariable cox-regression analyses in patient-derived scaffolds cultured with MDA-MB-231 following treatment with 5-fluorouracil and doxorubicin. **Table S13.** Table with P-values from log-rank tests of the Kaplan Meier plots illustrating disease-free survival in patient-derived scaffolds cultured with MDA-MB-231 and treated with 5-fluorouracil and doxorubicin.

## Data Availability

All data generated or analyzed during this study are included in this published article and its supplementary information files.

## Abbreviations

PDS: Patient-derived scaffolds
CSC: Cancer stem cells
EMT: Epithelial-to-mesenchymal-transition
5-FU: 5-Fluororacil
DOX: Doxorubicin
DMEM: Dulbecco’s modified Eagle’s medium
RPMI medium: Roswell Park Memorial Institute medium
IQR: Interquartile range
DFS: Disease-free survival
Q1: First quartile
Q3: Third quartile
ER: Estrogen receptor
PR: Progesterone receptor
LN Met: Lymph node metastases

## References

[1] Pecorino L. Molecular Biology of Cancer. Mechanisms, Targets, and Therapeutics. Oxford: Oxford University Press; 2012.

[2] Mortezaee K, Majidpoor J (2022). Key promoters of tumor hallmarks. Int J Clin Oncol.

[3] Bray F (2018). Global cancer statistics 2018: GLOBOCAN estimates of incidence and mortality worldwide for 36 cancers in 185 countries. CA Cancer J Clin.

[4] Nolan E, Lindeman GJ, Visvader JE (2023). Deciphering breast cancer: from biology to the clinic. Cell.

[5] Prasad P (2013). Doxorubicin and mitomycin C co-loaded polymer-lipid hybrid nanoparticles inhibit growth of sensitive and multidrug resistant human mammary tumor xenografts. Cancer Lett.

[6] Helleman J (2008). Association of an extracellular matrix gene cluster with breast cancer prognosis and endocrine therapy response. Clin Cancer Res.

[7] Farmer P (2009). A stroma-related gene signature predicts resistance to neoadjuvant chemotherapy in breast cancer. Nat Med.

[8] Finak G (2008). Stromal gene expression predicts clinical outcome in breast cancer. Nat Med.

[9] Tekpli X (2019). An independent poor-prognosis subtype of breast cancer defined by a distinct tumor immune microenvironment. Nat Commun.

[10] Sachs N (2018). A Living Biobank of Breast Cancer Organoids Captures Disease Heterogeneity. Cell.

[11] Landberg G (2020). Patient-derived scaffolds uncover breast cancer promoting properties of the microenvironment. Biomaterials.

[12] Parkinson GT (2021). Patient-derived scaffolds as a model of colorectal cancer. Cancer Med.

[13] Garre E (2022). Breast Cancer Patient-Derived Scaffolds Can Expose Unique Individual Cancer Progressing Properties of the Cancer Microenvironment Associated with Clinical Characteristics. Cancers (Basel).

[14] Persson E (2021). Patient-derived scaffolds influence secretion profiles in cancer cells mirroring clinical features and breast cancer subtypes. Cell Commun Signal..

[15] Leiva MC (2021). Breast cancer patient-derived scaffolds as a tool to monitor chemotherapy responses in human tumor microenvironments. J Cell Physiol.

[16] Gustafsson A (2021). Patient-derived scaffolds as a drug-testing platform for endocrine therapies in breast cancer. Sci Rep..

[17] Tilsed CM (2022). Cancer chemotherapy: insights into cellular and tumor microenvironmental mechanisms of action. Front Oncol.

[18] Anand U (2023). Cancer chemotherapy and beyond: Current status, drug candidates, associated risks and progress in targeted therapeutics. Genes Dis.

[19] Bustin SA (2009). The MIQE guidelines: minimum information for publication of quantitative real-time PCR experiments. Clin Chem.

[20] Kalluri R, Weinberg RA (2009). The basics of epithelial-mesenchymal transition. J Clin Invest.

[21] Mani SA (2008). The epithelial-mesenchymal transition generates cells with properties of stem cells. Cell.

[22] Morel AP (2008). Generation of breast cancer stem cells through epithelial-mesenchymal transition. PLoS ONE.

[23] Bhat-Nakshatri P (2010). SLUG/SNAI2 and tumor necrosis factor generate breast cells with CD44+/CD24- phenotype. BMC Cancer..

[24] Nader GPF (2021). Compromised nuclear envelope integrity drives TREX1-dependent DNA damage and tumor cell invasion. Cell.

[25] Yu Z (2012). Cancer stem cells. Int J Biochem Cell Biol.

[26] Li Y (2020). Matrix Stiffness Regulates Chemosensitivity, Stemness Characteristics, and Autophagy in Breast Cancer Cells. ACS Appl Bio Mater.

[27] Lovitt CJ, Shelper TB, Avery VM (2018). Doxorubicin resistance in breast cancer cells is mediated by extracellular matrix proteins. BMC Cancer..

[28] Insua-Rodríguez J (2018). Stress signaling in breast cancer cells induces matrix components that promote chemoresistant metastasis. EMBO Mol Med..

[29] Haro M, Orsulic S (2018). A paradoxical correlation of cancer-associated fibroblasts with survival outcomes in B-cell lymphomas and carcinomas. Front Cell Dev Biol..

[30] Giraldo NA (2019). The clinical role of the TME in solid cancer. Br J Cancer.

[31] Loh JJ, Ma S (2021). The Role of Cancer-Associated Fibroblast as a Dynamic Player in Mediating Cancer Stemness in the Tumor Microenvironment. Front Cell Dev Biol.

[32] Katayama MLH (2019). Stromal Cell Signature Associated with Response to Neoadjuvant Chemotherapy in Locally Advanced Breast Cancer. Cells.

[33] Zhu J (2015). Six stroma-based RNA markers diagnostic for prostate cancer in European-Americans validated at the RNA and protein levels in patients in China. Oncotarget..

[34] Sensi F (2020). Recellularized Colorectal Cancer Patient-derived Scaffolds as in vitro Pre-clinical 3D Model for Drug Screening. Cancers (Basel).

[35] Tonellotto F (2019). Impact of number of positive lymph nodes and lymph node ratio on survival of women with node-positive breast cancer. Eur J Breast Health..

[36] Min SK (2021). Relation Between Tumor Size and Lymph Node Metastasis According to Subtypes of Breast Cancer. J Breast Cancer.

[37] Gotz C (2018). ALDH1 as a prognostic marker for lymph node metastasis in OSCC. Biomed Rep..

[38] Durinikova E (2018). ALDH1A3 upregulation and spontaneous metastasis formation is associated with acquired chemoresistance in colorectal cancer cells. BMC Cancer..

[39] Marcato P (2011). Aldehyde dehydrogenase: its role as a cancer stem cell marker comes down to the specific isoform. Cell Cycle.

[40] Croker AK (2017). Differential Functional Roles of ALDH1A1 and ALDH1A3 in Mediating Metastatic Behavior and Therapy Resistance of Human Breast Cancer Cells. Int J Mol Sci.

[41] Horiguchi K (2010). Predictive value of CD24 and CD44 for neoadjuvant chemotherapy response and prognosis in primary breast cancer patients. J Med Dent Sci..

