# Supplementary material for: Patient-derived scaffolds representing breast cancer microenvironments influence chemotherapy responses in adapted cancer cells consistent with clinical features
Source: J Transl Med. 2023 Dec 20;21:924. doi: 10.1186/s12967-023-04806-z (PMC10734148; doi:10.1186/s12967-023-04806-z)
Supplement: Supplementary file 1 — Additional file 1: Fig. S1. RNA yield in MCF7 patient-derived scaffold lysates. Fig. S2. Significant associations between 5-flourouracil drug fingerprints and clinical characteristics of the original tumors. Fig. S3. Significant associations between doxorubicin drug fingerprints and clinical characteristics of the original tumors. Fig. S4. Kaplan–Meier plots displaying the relationship between disease-free survival and drug fingerprints in patient-derived scaffolds. Fig. S5. RNA yield in MDA-MB-231 patient-derived scaffold lysates. Fig. S6. Drug fingerprints of patient-derived scaffold cultures with MDA-MB-231. Fig. S7. Scatter plots for clinical characteristics versus MDA-MB-231 drug fingerprints. [file 12967_2023_4806_MOESM1_ESM.pdf]

Additional figures

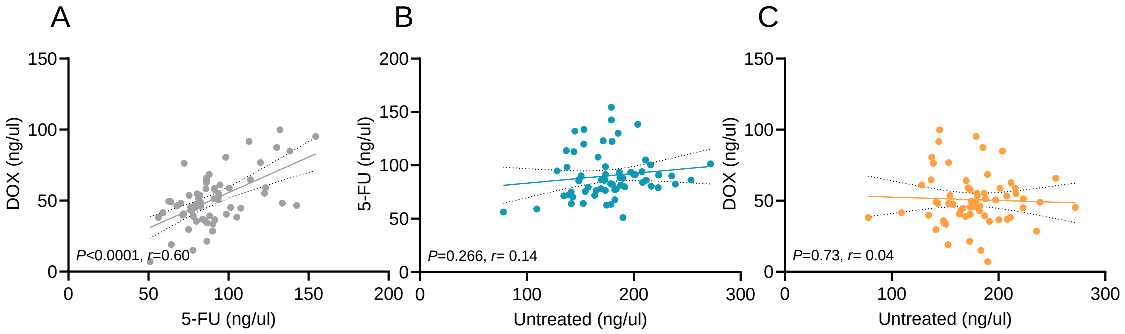

Additional Figure 1. RNA yield in MCF7 patient-derived scaffold lysates. Pearson correlation analyses of RNA yield (ng/µl) from A) doxorubicin (DOX) versus 5-flourouracil (5-FU) treated patient-derived scaffolds (PDSs). B) 5-FU treated versus untreated PDSs. C) DOX treated versus untreated PDSs. *P*<0.05 were considered significant, *r*= Pearson correlation coefficient.

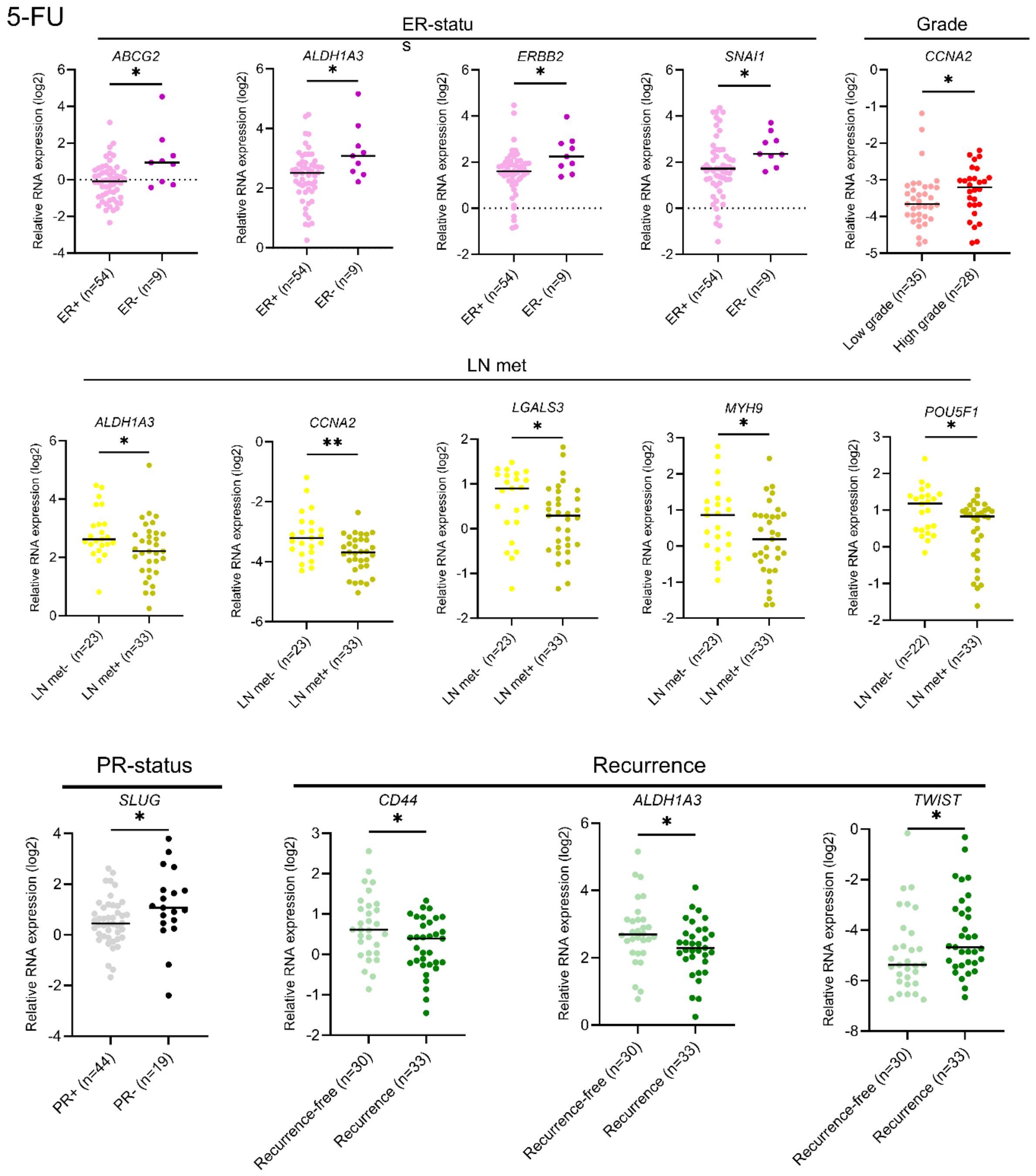

Additional Figure 2. Significant associations between 5-flourouracil drug fingerprints and clinical characteristics of the original tumors. Induced gene expression changes in MCF7 cells after treatment with 5-fluorouracil (5-FU) in relation to estrogen receptor status (ER), grade, lymph node metastasis (LN met), progesterone receptor (PR) and recurrence of breast cancer or metastatic disease in patients (**P*<0.05, ***P*<0.01, Mann-Whitney *U*)

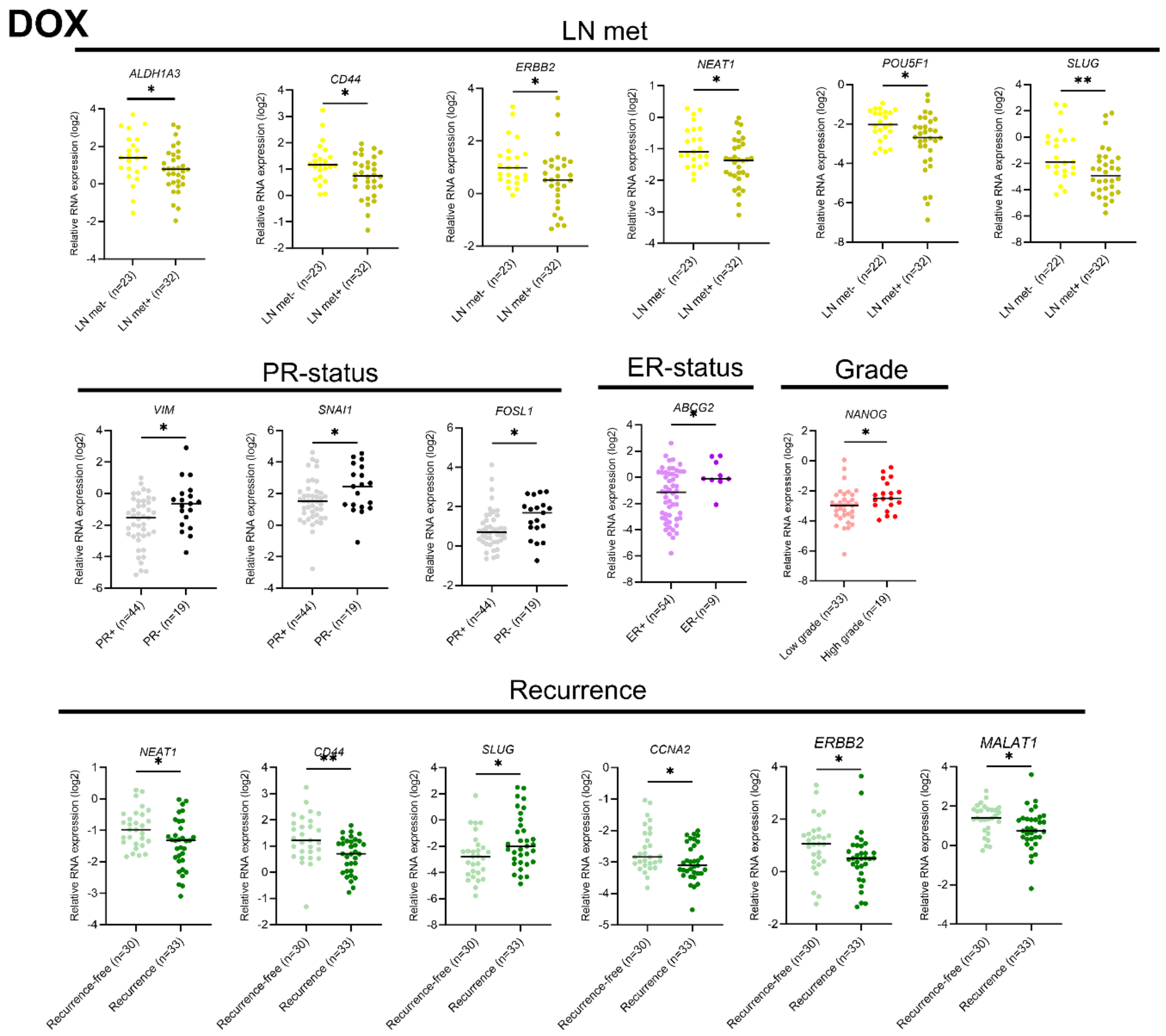

Additional Figure 3. Significant associations between doxorubicin drug fingerprints and clinical characteristics of the original tumors. Induced gene expression changes in MCF7 cells after treatment with doxorubicin (DOX) in relation to grade, lymph node metastasis (LN met) progesterone receptor (PR) and estrogen receptor status (ER), recurrence of breast cancer or metastatic disease in patients (**P*<0.05, ***P*<0.01, Mann-Whitney U)

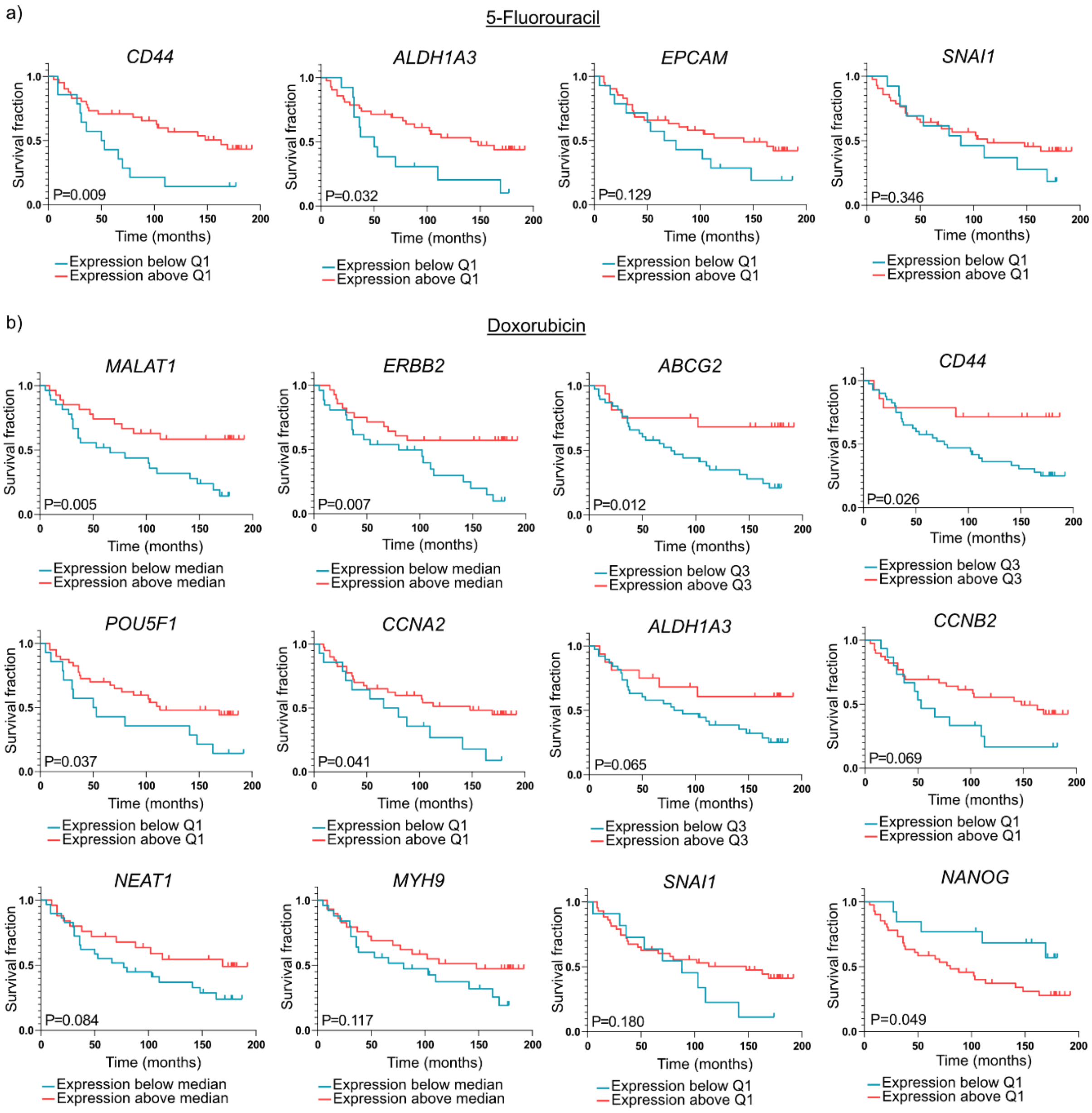

Additional Figure 4. Kaplan-Meier plots displaying the relationship between disease-free survival and drug fingerprints in patient-derived scaffolds. Kaplain-Meier analyses displaying the stratification of disease-free survival of patient having the original tumors based on their induced high (red) or low (blue) PDS drug fingerprints of genes identified in the multivariable analyses for A) 5-fluorouracil or B) doxorubicin treated PDSs. **P*<0.05, log-rank test (detailed information in Additional Table 8).

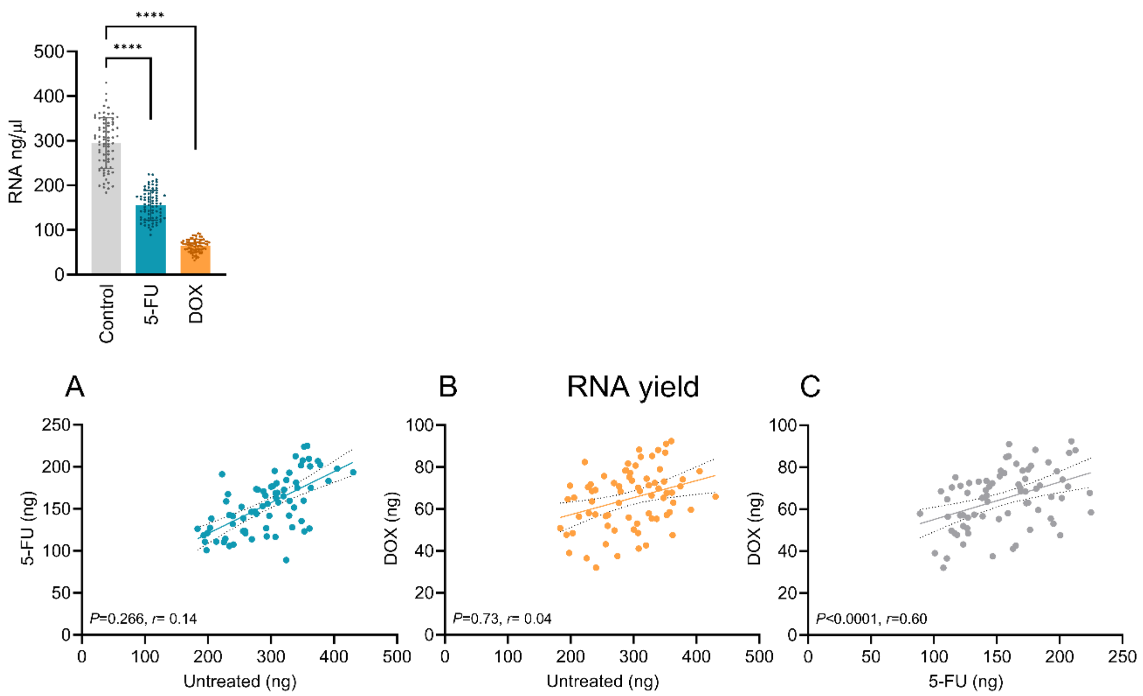

Additional Figure 5. RNA yield in MDA-MB-231 patient-derived scaffold lysates. A) RNA yield (ng/µl) in lysates from MDA-MB-231 patient-derived scaffold (PDS) cultures after 21 days of growth for controls, 5-fluorouracil (5-FU) or doxorubicin (DOX) treated samples. (****P<0.0001, untreated versus treated PDSs. Two-Way Anova with Dunnett's multiple comparisons test). B, C, D) Pearson correlation analyses of RNA yield (ng/µl) from DOX versus 5-FU treated PDSs (B), 5-FU treated versus untreated PDSs (C) and DOX treated versus untreated PDSs (D). P<0.05 were considered significant, r= Pearson correlation coefficient.

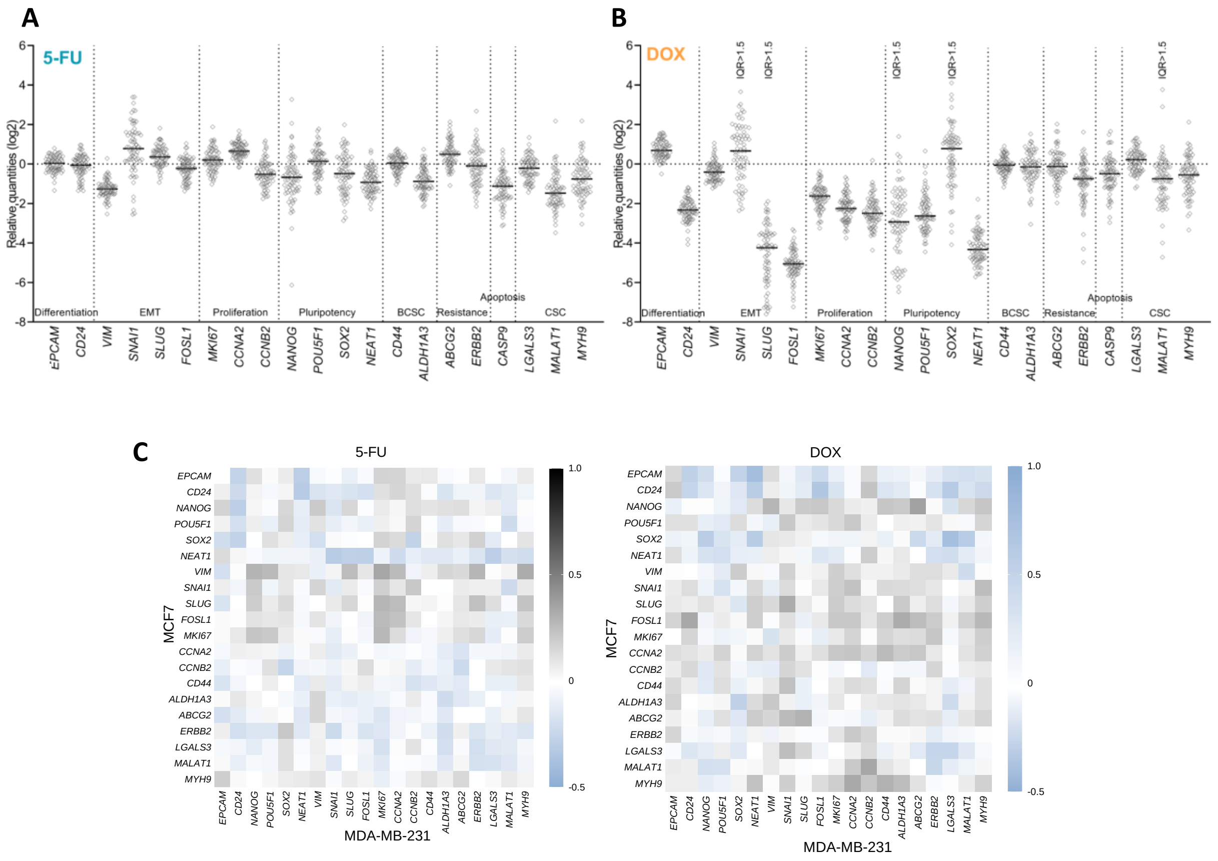

Additional Figure 6. Drug fingerprints of patient-derived scaffold cultures with MDA-MB-231. Drug fingerprints of MDA-MB-231 PDS-cultures where, for each gene, the untreated value (control) is subtracted from the treated value after A) 5-fluorouracil (5-FU) or B) doxorubicin (DOX). The largest variation in gene expression changes between individual PDSs is indicated by interquartile range> 1.5 (IQR. Ratio of PDS-fingerprints between quartile 1 to 3). Gene expression data is expressed in log2-scale. C) Heatmaps showing the Pearson correlation analyses between the drug fingerprints for MCF7 versus MDA-MB-231 in 58 overlapping PDSs after 5-FU treatment or DOX treatment. Scale bar displays the Pearson correlation coefficient (*P*-values for each analysis are detailed in Additional Table 10).

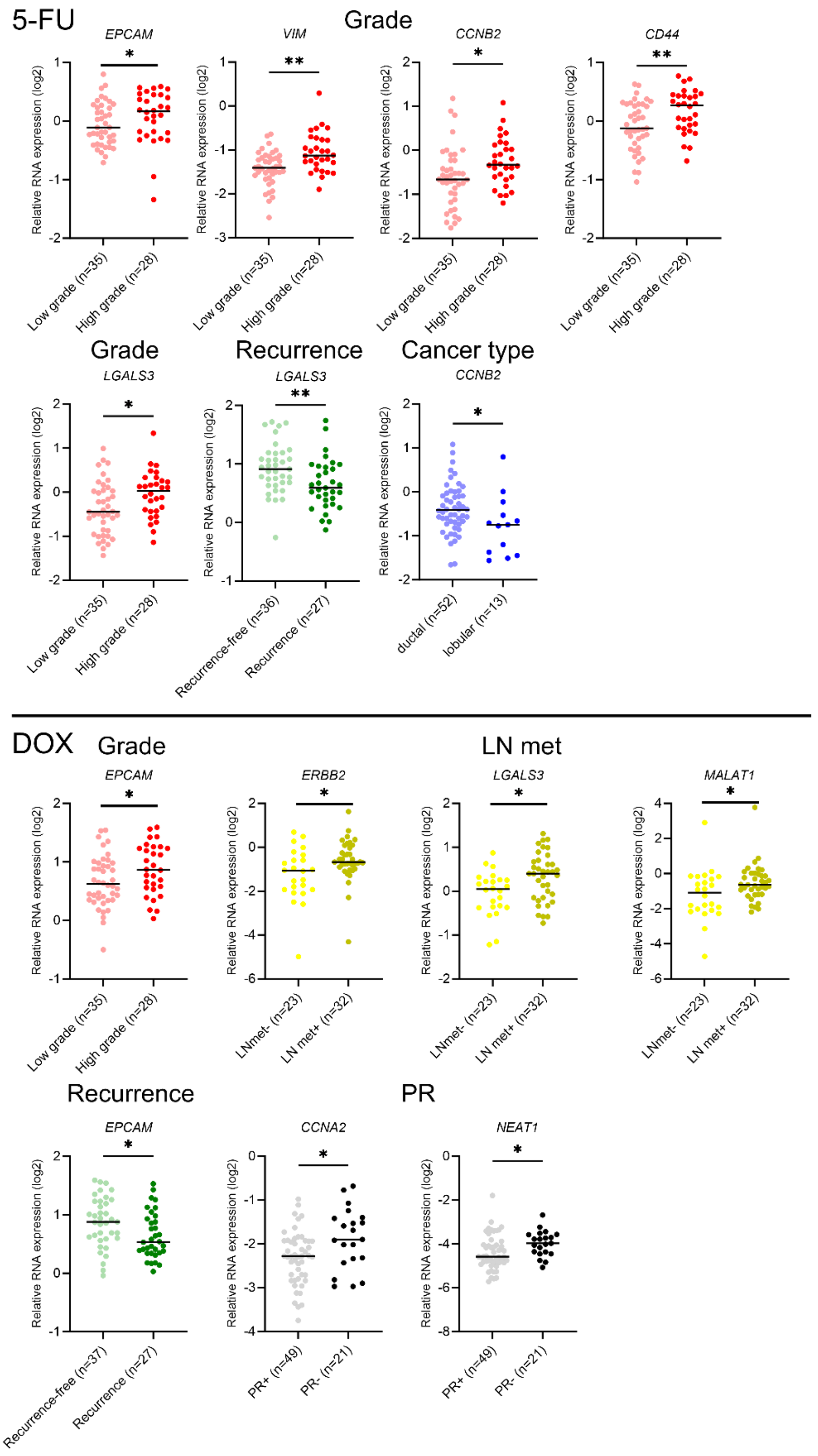

Additional Figure 7. Scatter plots for clinical characteristics versus MDA-MB-231 drug fingerprints. Induced gene expression changes in MDA-MB-231 cells after treatment with 5-fluorouracil (5-FU) and doxorubicin (DOX) in relation to grade, lymph node metastasis (LN met), progesterone receptor (PR) and estrogen receptor (ER), and recurrence-status (**P*<0.05, ***P*<0.01, Mann-Whitney U).
